# Patient Satisfaction Determinants of Inpatient Healthcare

**DOI:** 10.3390/ijerph182111337

**Published:** 2021-10-28

**Authors:** Beata Gavurova, Jan Dvorsky, Boris Popesko

**Affiliations:** Faculty of Management and Economics, Tomas Bata University in Zlín, Mostní 5139, 760 00 Zlín, Czech Republic; j1dvorsky@utb.cz (J.D.); popesko@utb.cz (B.P.)

**Keywords:** patient, satisfaction, hospitalisation, healthcare, healthcare quality, health policy

## Abstract

The aim of the study was to analyse and evaluate the determinants influencing the overall satisfaction of patients with inpatient healthcare in the conditions of the Czech Republic. A total of the 1425 patients, who experienced hospitalisation and agreed to participate, were questioned in the study. A research questionnaire was used to obtain data on satisfaction with hospitalisation. The subject of the research consisted of the indicators related to the following factors: (i) satisfaction with the hospital, clinic, room and meals; (ii) satisfaction with medical staff—nurses, physician expertise and other staff; (iii) the quality of the treatment provided; (iv) satisfaction with leaving the hospital. The formulated statistical hypotheses were evaluated through structural equation modelling. The results of the analyses brought interesting findings. Satisfaction with medical staff is the most significant factor which has a positive effect on satisfaction with hospitalisation. Physician expertise (with trust and good communication skills) is more important for patients than satisfaction with nurses or other staff. The results obtained from the study represent valuable information for policymakers, regional healthcare plans, as well as for managers of hospitals.

## 1. Introduction

Patient satisfaction is considered an important part of the healthcare quality assessment. Over the last decades, the diverse systems for measuring patient satisfaction have been developed gradually, with their structure and complexity depending on several aspects that have been monitored and evaluated in terms of patient satisfaction. Some authors distinguish between the two aggregate forms of patient satisfaction: technical quality and functional quality [1,2,3]. They define technical quality through the technical accuracy of the diagnosing and treating procedures. Functional quality refers to the way in which healthcare is provided to a patient, for instance, how it is linked to the way the diagnostic or therapeutic procedures are performed. As the demands on the quality of healthcare increase, so the demands for monitoring and evaluating patient satisfaction are amplified too, and it has supported the development of the policies to monitor patient satisfaction [4].

The differences in the approaches of measuring and evaluating patient satisfaction in individual countries can also be seen between the different types of healthcare providers and are influenced by the different financial mechanisms, health policy, the structure of the health system in the country and according to the similar factors [5,6,7]. The private providers regularly measure patient satisfaction and transform their results into performance indicators. These performance indicators are part of their strategic goals as well as the regional health plans [8,9,10].

Patient satisfaction makes it possible to measure the consistency between patient preferences, expectations and the healthcare provided. Knowledge of information about the possibilities of increasing patient satisfaction and, thus, increasing the healthcare quality enables the creation of a competitive advantage for the healthcare facilities [11]. As patient satisfaction increases, trust is also built between the patient and the medical staff that helps to build loyalty bonds gradually and to increase the satisfaction of medical staff with their work [12,13]. Patient satisfaction thus becomes an essential part of a complex healthcare quality system.

Patient satisfaction is not a static indicator because it is affected by changes in the external environment of the health systems. As an example, we can cite the impact of the changes in healthcare availability. Elimination of the barriers to access to health care (financial, geographical, institutional) will increase the use of outpatient and inpatient healthcare services (ambulatory and hospital levels) that may subsequently be reflected in lower quality of the healthcare provided resulting from the limited human resources in the healthcare sector in the country or the region. Even the low affordability of healthcare services can have a negative impact on patient satisfaction [3,11,14]. Explicitly determining the significance of the factors that indicate patient satisfaction is not easy. Their preference in the overall assessment of patient satisfaction depends on the expected usability of the results aimed at improving processes in healthcare facilities. (Vocabulary as an aid: communication, showing courtesy towards patients and an environment of the facility significantly predicts patients’ satisfaction with the quality of healthcare.)

Some research studies have examined the changes in the patient satisfaction indicators levels in relation to the introduction of the different healthcare programmes or in connection with the reorganisation of the healthcare facilities’ network, the introduction of innovative processes or the modifications in the treatment and diagnostic procedures. Obtaining relevant outputs, which quantify the changes in healthcare through patients, requires setting up quality data collection primarily and creating the specific data systems that will not only evaluate the planned aspects but also reveal other contexts (secondary effects) that may affect the quality of healthcare through the patient satisfaction. The research studies play an important role in this aspect, and they enable sharing the research findings between the countries and regions and the creation of their own measurement tools for measurement and evaluation of patient satisfaction [6,14]. These tools will make it possible to improve, from a microeconomic point of view, the efficiency of the healthcare facilities and, from a macroeconomic point of view, the sustainability of the health systems in individual countries [10,15]. At the same time, effective tools to support the improvement in the quality of health care in the healthcare facilities provide a platform for the development of relevant policies and for the improvement in the healthcare systems [9,16,17].

## 2. Background of the Study

An examination of patient satisfaction has long been the subject of multidisciplinary research, in which we see a strong synergy of the results. The complexity of the diagnostic, as well as treatment procedures and the belief in their success, increase the patients’ expectations, while they do not only evaluate this satisfaction from a medical point of view. For the evaluation of patient satisfaction, the comprehensive quality of the provided healthcare is becoming increasingly important, and the environment in which the healthcare is provided and patient recovery during treatment is also evaluated. This clearly implies the complexity of measuring and evaluating patient satisfaction related to the methodological complexity as well as access to the more deeply structured data. The results of many research studies conducted in recent years provide us with valuable insights into the possibilities of improving the quality of healthcare by increasing patient satisfaction [18,19,20]. Patient satisfaction is influenced by several factors, and the subjective perception of satisfaction makes it difficult to compare and unify the outcomes [21,22,23].

Farzianpour et al. [16] considered patient satisfaction as the cognitive response that is influenced by various factors. They considered the determination of the factors influencing patient satisfaction as an indirect way to achieve real patient satisfaction. The authors emphasised that in addition to a determination of the factors influencing patient satisfaction, understanding the expectations and needs of the patients is also an important fact because it is possible to quantify the deviations from the real situation from an expected state. Their knowledge will enable the healthcare facilities to eliminate the causes of dissatisfaction effectively and, thus, increase the level of the healthcare services provision gradually. In a competitive healthcare environment, patient satisfaction with their quality of life is more and more linked. Patient satisfaction is also affected by the treatment processes, which the patient with different diagnoses and comorbidities may manage differently [24,25,26,27]. Consistency of the treatment and healthcare processes in relation to patient vulnerabilities and the need for comprehensive support is important.

Patient satisfaction is the result of a complex set of various factors, including coordination of the various aspects of the services, such as medicine, nursing, services from the various organisational sections, and so forth, whilst it is necessary to fully respect the patient rights in all the aspects and to create the optimal conditions for healthcare services improvement. Farzianpour et al. [16] reviewed numerous research studies from recent years to provide a clear picture of the overall patient satisfaction and the main determinants that affect it. They pointed out that the results of the different studies in this area are quite inconsistent. Santuzzi et al. [17] identified several factors effective in analysis and evaluation of satisfaction: age, gender, marital status, level of education, patient social status, waiting time for services, hospital staff skills, services provided by physicians and nurses, providing instructions to patients during release, respecting the opinions of patients, the state of patient insurance and so on. The authors stated that it is important to pay attention to the research sample of patients and to examine in detail the reasons for patient dissatisfaction that can provide valuable information for managers and planners of healthcare facilities. Bjertnaes et al. [28] pointed out that it is important to include in the evaluation of the healthcare quality not only patient satisfaction and experience but also their expectations. The patient expectations are only rarely included in the evaluation systems. The authors attempted to estimate the effects of the various predictors of overall patient satisfaction with hospitalisations, including the patient-reported experiences, meeting patient expectations and the socio-demographic variables. The most important predictors were patient-reported experience, followed by compliance with the patient expectations, the physician experience and the perceived improper treatment. Age was not a significant predictor of overall patient satisfaction. Marimon et al. [29] examined with the patient expectation parameters in their study. The authors stated that despite the numerous efforts to define the scale of quality measurement in the healthcare provision, there is no complete agreement on which determinants affect it the most, and there is a lack of quality scales that would capture all the patient concerns related to hospitalisation. The authors attempted to define a scale called HospQual to assess the perceived quality of the hospital services by patients and analyse the impact of perceived quality on patient satisfaction. They showed that meeting expectations is a key link between quality and satisfaction.

Open narrative reviews of the patients in social media can also be an important source of information to shed light on the important aspects of patient satisfaction in hospitals. This aspect was verified in the research study by Chakraborty and Church [29,30,31]. This is a unique study, where the authors used the qualitative and quantitative methods to evaluate the comments on the reviews of hospitalisations of the respondents that were freely made public on the social networks. The authors critically evaluated which factors are most important for patient satisfaction assessment. The healthcare providers should regularly analyse comments on social networks from patients to help the health teams to understand the critical aspects of patient perception of the quality of the healthcare processes. By regular analysis of patient comments on social media, the hospital managers can quickly identify and address the shortcomings in the provision of healthcare service that could ultimately give the hospital a significant competitive advantage.

The significance of social networks for patient satisfaction assessment of hospitalisation bears importance for the healthcare service providers as well as for the health policymakers, and this is confirmed by the latest studies [32,33,34]. Chakraborty and Church [31] emphasised the importance of social media assessments as an effective complement to obtain information from Hospital Consumer Assessment of Healthcare Provider and Systems (HCAHPS), for which the hospital managers have to wait a year at least. In this way, they can eliminate the problems reported by patients on a regular basis. Rastegar-Mojarad et al. [35] saw a problem in the more intensive use of social media in a case of the absence of patient reviews that may cause a major bottleneck in applying computational techniques in the future.

Geographical aspects act as a common differentiating factor in an examination of the determinants of hospitalised patient satisfaction. Liu et al. [36] examined the satisfaction of hospitalised patients at the district level, whilst their research sample consisted of 1458 adult hospitalised patients. The analysis outcome showed that the patient and institutional characteristics were strongly associated with inpatient satisfaction. The patients with higher educational levels were more satisfied with the administrative process. The satisfaction differences were also evident in the age and sex aspects, and according to the localities: elder patients and patients with worse self-reported health status were less satisfied with the hospital environment. The patients receiving care in suburban hospitals were less satisfied with the administrative process, the hospital environment and the overall satisfaction. However, chronic disease and hospital grade were not significantly associated with satisfaction in all the examined domains. Kraska et al. [37] also drew attention to the absence of research on the impact of hospital characteristics on patient satisfaction. The authors examined the four dimensions of patient satisfaction: medical care, nursing care, organisation and overall impression, which were analysed as the outcome measures of the research. The region, profit orientation, size, staffing per bed and quality scores were considered as possible influencing hospital characteristics. All the analysed variables had a significant effect on the patient satisfaction dimensions, but the differences in patient satisfaction were found in the hospitals located in the different locations. Patients were more satisfied in small hospitals, in non-profit hospitals, in hospitals where there was more medical staff per bed and where the process and outcome quality was associated with the more satisfied patients. These findings confirm that the patients are sensitive to important hospital quality measures. Mann et al. [38] drew attention to the fact that it is important to examine the effects of survey results, such as HCAHPS, differentiated according to individual factors. They pointed out that the satisfaction determinant of physician communication with the patient has not improved for a long time in all the hospitals (data from HCAHPS 2007–2013). The overall gap between the hospitals has narrowed, which can be further improved through sharing the best practices. Davidson et al. [39] drew attention to the fact that there have long been efforts by many hospital systems in countries to improve patient satisfaction, as stated by the HCAHPS surveys. Although many studies have shown some improvement in the HCAHPS scores through various interventions, more rigorous research will be needed in the future to identify effective and generalisable interventions.

Many research studies expected the usefulness of the patient satisfaction assessment results for health literacy improvement processes as well. Weidmer et al. [40] stated that the results of HCAHPS can serve not only as a tool for quality improvement but also to measure whether the healthcare providers in a hospital setting have communicated effectively with their patients. In this regard, Brega et al. [41] emphasised the importance of an examination of Organizational Health Literacy (OHL). According to the authors, the OHL is the degree to which health care organisations implement strategies to make it easier for patients to understand health information, navigate the health care system, engage in the health care process and manage their health. The authors critically pointed out that only a few measures that organisations can use to monitor their improvement efforts have been implemented in this area. Bremer et al. [42] sought to facilitate research in the field of organisational health literacy by answering the question, which criteria characterise a health literate health care organisation. The OHL involves a large number of the different organisational criteria that make standardisation as well as comparability considerable. The terminology applied in OHL is highly heterogeneous, and it is based on different concepts. The OHL comprehensive conceptual framework, based on consensus, is still absent. This is also a challenge for a deeper examination of its impact in relation to increasing patient satisfaction. Siddiqui et al. [43] drew attention to the issue of the non-uniform comparative basis on patient satisfaction assessment in the different types of hospitals that can create misleading ideas about the differences in satisfaction in them. In their research, the authors compared patient satisfaction in acute-care hospitals and general medical hospitals. The authors found that the different results between the different types of hospitals lie in the survey response rate and the subdomains of patient satisfaction. The results of the study by Predkiewicz et al. [7] reflected similar issues. The methodological aspects of the comparative analyses and the size of the research sample are still very important factors for the evaluation processes. All the above consistent facts justify the importance of examining the determinants of patient satisfaction in healthcare facilities that will improve the quality of healthcare provided and, thus, the efficiency of the country’s health system.

This was also the motivation for the implementation of our research, the aim of which was to analyse and evaluate the determinants influencing the overall satisfaction of patients with inpatient healthcare in the conditions of the Czech Republic.

## 3. Materials and Methods

### 3.1. Data Collection and Questionnaire

The data collection was carried out from September 2020 to January 2021. A patient was defined as a person who was hospitalised at least once in their life in a hospital in the Czech Republic. Patient participation in the satisfaction evaluation with inpatient healthcare service was voluntary (the patient had the option not to provide attitude). The subject of the evaluation was the last hospitalisation of the respondent. Several forms of inquiry were applied through the questionnaires for data collection, namely filling in a questionnaire in a paper version at the hospital department directly, filling in a questionnaire in an electronic version through a request to fill in the questionnaire. The results of the power analysis demonstrated that the sample size of the respondents is at a sufficient level. The questions in the questionnaire were formulated into individual sections (demographic (age, gender, education), economic (net monthly income of the respondent, number of persons in the household), social (e.g., marital status), or healthcare (e.g., disability, alcohol, spirit, smoking) questions on the respondent, the perception of the quality of inpatient healthcare and so on. When creating the content of the research questionnaire, we were inspired by the research in the HCAHPS methodologies with a reflection on the specifics of the individual countries in which the research was conducted and the health systems that were used in these countries. We also used experience from our own research activities and cooperation with the Ministry of Health of the Czech Republic, the Ministry of Health of the Slovak Republic, the Institute of Health Policy of the Slovak Republic, and we also used experience from our own expertise to evaluate the quality of service in healthcare facilities in the Czech Republic and the Slovak Republic. A total of 1488 responses were obtained, of which 1425 (95.8%) were filled in correctly, and 63 (4.2%) were filled in incorrectly. The most common reasons to exclude the responses from the respondents were: unconfirmed consent to the publication of patient attitudes, incorrect demographic data about the patient (for instance, the year of birth 1470, age of the respondent 160, a number of the members in the household 30 and so forth). The questionnaire also included a control question to verify the consistency of the evaluation of the patient’s hospitalisation health care. The questionnaire could be filled in: patient—1143 (80.2%); patient with the help of a close person—59 (4.1%); patient with the medical assistance—32 (2.3%); friend/girlfriend or relative of the patient (if the patient was not over 18 years old)—191 (13.4%).

A total of 1425 respondents were involved in the research. The structure of the research sample was as follows: gender—male/female: 556/869 (39.0%/61.0%); age—up to 30 years old/over 30 years old: 899/526 (63.1%/36.9%); marital status—single/other (married, widowed, divorced, in registered partnership): 942/483 (66.1%/33.9%); education level—primary and secondary school without General Certificate of Secondary Education/secondary school/university: 219/884/322 (15.4%/62.0%/22.6%); monthly income (in EUR)—0–456/457–760/more than 760 EUR: 642/279/504 (45.0%/19.6%/35.4%); monthly income of the whole household (in EUR)—0–1330/1331–2090/more than 2090 EUR: 443/464/518 (31.1%/32.6%/36.4%); number of household members—0–2/3/4 and more: 450/389/586 (31.6%/27.3%/41.1%); number of dependent children—0/1/2/3 and more: 338/362/449/276 (23.7%/25.4%/31.5%/19.4%); smoking—regularly/occasionally/in the past/not: 168/287/173/797 (11.8%/20.1%/12.1%/55.9%).

### 3.2. Methods

A multidimensional method of structural equation modelling (SEM) was applied to estimate and verify the relationship between the variables. SEM is a combination of two statistical methods: Confirmatory Factor Analysis (CFA) and Path Analysis (PA) [44]. The aim of the CFA method was to estimate the latent traits, such as attitude and satisfaction of patients on selected factors (LVs) and their items (MVs). The aim of the PA method was to find the causal relationship variables (LV1, ..., LV5) by creating a path diagram—“SEM model” (). The benefit of the SEM model is to find and quantify statistically significant (direct, indirect) relationships among multiple variables (LVs, MVs). Several authors point out that in order to apply the SEM method, the normal distribution assumption of the selected variables has to be met [45,46,47]. For this purpose, the basic descriptive characteristics were calculated. These served to compute the z-score values. The standard deviation and skewness were found to be in the range of ±1.5 and the kurtosis values in the range of ±3. Our data showed a pattern of normality [48].

According to Hair et al. [49], the CFA method is able to evaluate and confirm constructs (LVs) and indicators (MVs), which have already been formulated in the theoretical section of the paper. The CFA approach determines the importance of the implemented items and components, whether they are compatible with the study performed (e.g., [50]). The authors applied a rotated component matrix to analyse factor loadings, extracted mean variances and composite reliabilities. The Kaiser–Meyer–Olkin (KMO) test and the Bartlett test of sphericity were used to verify the suitability of the data [47,51]. If the KMO value is lower than 0.5, it does not make sense to perform a factor analysis [49]. Principal Component Analysis (PCA) was used to extract the factors. A network graph [52] and a matrix of components were applied to decide the number of factors. The Varimax orthogonal rotation method was used to rotate the factors [53].

The suitability of the SEM model was verified using the Fit test summary (based on [54,55,56,57]): Goodness of Fit (GFI); CMIN/DF—The minimum discrepancy; Comparative Fit index (CFI); Root Mean Square Error of Approximation (RMSEA); Normed fit index (NFI). The significance level was 5% (α = 0.05). The evaluation of statistical hypotheses was performed in IBM SPSS Statistics and IBM SPSS Amos. Graphical visualisation of relationships between variables (model) was performed in IBM SPSS Amos (IBM Inc., Armonk, NY, USA).

### 3.3. Variables

The following latent variables (LVs, the independent variables: LV1, LV2, LV3, LV4; the dependent variable: LV5) and the manifest variables (MVs) were the subject of the statistical investigation:

- Satisfaction with hospital, department, room and board (LV1): MV_11—waiting time for room allocation from the moment the patient came to the hospital (type of answer (TA): ten-point scale (from I waited very long—0 to I did not wait at all—10); MV_12—patient harassment due to night noise of other patients (TA: yes—0; no—1); MV_13—patient harassment due to night noise of medical staff (TA: yes—0; no—1); MV_14—cleanliness of the hospital room and ward (TA: ten-point scale from not clean at all—0 to very clean—10); MV_15—choice of meal in the hospital (TA: no—0; yes, sometimes—1; yes, always—2).
- Satisfaction with medical staff (nurses, doctor’s expertise, nurses and other staff) (LV2): MV_21—assessment of trust in the attending physician (TA: ten-point scale from worst trust—0 to best trust—10); MV_22—communication of the physician in front of the patient as if the patient was not present there (TA: yes—0; no—1); MV_23—assessment of trust in the nurse (TA: ten-point scale from worst trust—0 to best trust—10); MV_24—communication of medical staff in front of the patient as if the patient was not present there (TA: yes—0; no—1); MV_25—sufficient number of nurses who cared for patients (ten-point scale since there was never a nurse—0 after the maximum number of nurses was always—10); MV_26—awareness of the exchange of work shifts of nurses taking care of the patient’s healthcare (TA: no—0; sometimes—1; yes—2).
- Satisfaction with the quality of the treatment provided (LV3): MV_31—evaluation of collaboration between the members of the nursing staff (TA: ten-point scale from no cooperation—0 to the best cooperation—10); MV_32—difference in the provision of information to the patient by the medical staff for the same thing (TA: ten-point scale from it regularly happened to me—0 to never happened to me—10); MV_33—involvement of the patient in the decision making process about their treatment to the extent that the patient would require (TA: ten-point scale from no involvement—0 to the highest involvement—10); MV_34—patient confidence in the decisions about health or treatment (TA: ten-point scale from no trust—0 to the greatest trust—10); MV_35—evaluation of the availability of information about the patient’s health condition or treatment (ten-point scale from I did not receive any information—0 to I received sufficient information—10); MV_36—assessment of the comprehensibility of information about the patient’s health condition or treatment from medical staff (ten-point scale from were not at all comprehensible—0 to were completely comprehensible—10); MV_37—evaluation of sufficient privacy when communicating about the health condition or treatment of the patient (ten-point scale from I had no privacy—0 to I always had sufficient privacy—10).
- Satisfaction with release from the hospital (LV4): MV_41—satisfaction with informing the patient about the date of hospital release (TA: ten-point scale I was not satisfied at all—0 after I was very satisfied—10); MV_42—patient release delay on the day when they should have been released for any reason (TA: yes—0; no—1); MV_43—written information to the patient about what the patient should/should not do after their release from the hospital (TA: no—0; yes—1).
- Satisfaction with inpatient care (LV5): MV_51—overall feeling of the patient that he was treated in the hospital with respect and dignity (TA: ten-point scale from I had a bad feeling—0 to I had a great feeling—10); MV_52—evaluation of the overall care in the hospital (TA: ten-point scale from the care was at an unsatisfactory level—0 to the care was at an excellent level—10); MV_53—patient request to evaluate the quality of the patient’s treatment during the hospital stay (TA: no—0; yes—1); MV_54—informing the patient by the hospital about how to proceed in a case of a complaint about the healthcare quality (TA: no—0; yes—1); MV_55—evaluation of healthcare by the other hospital staff (e.g., cleaners, concierge, kitchen staff and so on) (TA: ten-point scale from I had a bad feeling—0 to I had a great feeling—10). Selected factor, as is satisfaction with the hospital, department, room and board (LV1; hypothesis (H)–(H1); satisfaction with medical staff (LV2; H2); satisfaction with the quality of the treatment provided (LV3; H3) and satisfaction with release from the hospital (LV4; H4) has a statistically significant effect on satisfaction with inpatient care (LV5).

## 4. Research Results

### 4.1. Reliability and Validity Analysis

Table 1 does not contain all the examined MVs. The following ones did not meet the corrected item assumption. Total correlation (CI−TC): MV_12 (CI−TC = 0.324); MV_15 (CI−TCCI−TC = 0.490); MV_22 (CI−TC = 0.164); MV_26 (CI−TC = 0.054); MV_32 (CI−TC = 0.221); MV_43 (CI−TC = 0.379); MV_53 (CI−TC = 0.383); MV_54 (CI−TC = 0.421), because the values were less than 0.5. In addition, the FLs of MVs were less than 0.5. Therefore, the abovementioned MVs were not subject to further investigation. The results of CA and CR in Table 1 showed the fulfillment of reliability. The findings of Table 1 further demonstrated that FL of all the LVs was in the range of 0.50 to 0.93, which means that it meets the discriminant validity [48]. The AVE was more significant than 0.50 [48], which fulfils the constructs convergent validity criterion.

### 4.2. Kaiser–Meyer–Olkin (KMO) and Bartlett’s Analyses

The KMO analysis demonstrated the suitability and fitness of the data that presented the value of 0.956. According to Kaiser [47], it is at an excellent level because it considers the values in the range of 0.80 to 0.99. The outcomes of Bartlett’s Sphericity (Approx. Chi-Square = 12,884,344; degree of freedom. = 153; *p*-value = 0.000) exhibited the *p* < 0.05. This indicates that the correlation between the items was significant, and it was statistically significant at the five-per cent level of statistical significance (e.g., [58]). The results of commonalities were as follows: MVs—Initial = 1.000; Extraction (PCA) = values were higher than 0.500 (by all MVs).

### 4.3. Total Variance Explained—TVE

The cumulative variances of the latent variables pointed to a discrepancy in the variations of the probable variables. As the cumulative eigenvalue was higher than 1, the expected difference between the components was further established [59]. The outcomes of total variance also demonstrated a cumulative variance which was 66.1% (Initial Eigenvalues: LV5 = 45.016 %; LV1 = 6.293 %; LV2 = 5.371 %; LV3 = 4.900 %; LV4 = 4.507 %). This is considered to be quite good because the bottom threshold lay at 50 %. Therefore, based on cumulative eigenvalues and cumulative variance, the data sample is reliable, and it is ready for further analysis.

### 4.4. Structural Equation Modelling—SEM

**Figure 1:**
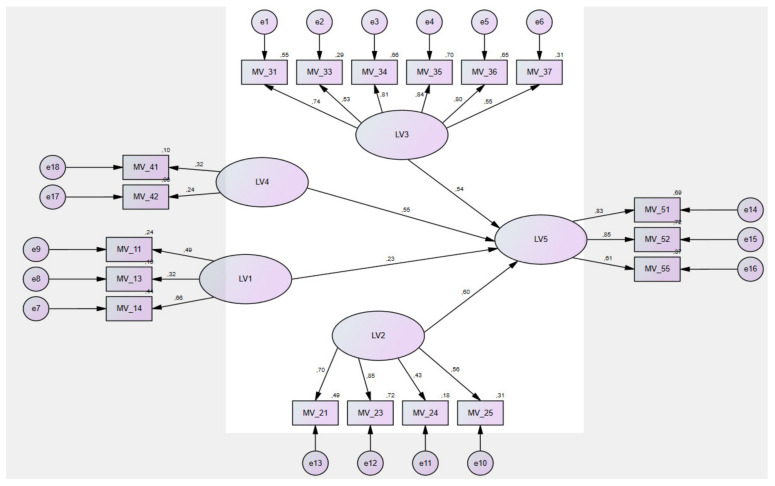
Final SEM model. Source: own research.

The maximum likelihood method was applied to estimate the parameters. The final SEM model of patient satisfaction with inpatient care consisted of the five latent variables (LV1, ..., LV5) and the eighteen manifest variables (MV_11, ..., LV55). The final SEM model and the relationships between the variables are shown in Figure 1.

The final SEM model (Figure 1) was the best solution between the measurement and structural model. Before evaluating and interpreting the structural model of relationships, calculations and evaluations of the most important FIT tests of the created SEM model were performed (Table 2; the number of distinct sample moments: 189; the number of distinct parameters to be estimated: 57; degrees of freedom: 132; CMIN = 3,465,327).

According to Hair et al. [49], the outcomes of Table 2 demonstrate that all the fit-indices results were within the specified range for the measurement model. Finally, it was concluded that a measurement model for patient satisfaction with inpatient care was appropriate.

### 4.5. Hypothesised Direct Relationship

Table 3 presents the results of direct effect applying the standardised regression weights between the four latent variables (LV1, ..., LV4) and satisfaction with inpatient care provided (LV5). The findings of Table 3 showed that framed hypotheses from H1 to H4 are supported since the probabilities’ T-values were less than the level of significance.

## 5. Discussion

The results of our analyses brought interesting findings. It was shown that the strongest factor with a direct impact on patient satisfaction with inpatient care was satisfaction with healthcare professionals, namely physicians, nurses, as well as other staff (β = 0.597). This factor was formed from the patient’s trust in the physician, from the patient’s trust in the nurse, from a sufficient number of nurses providing care for the patient, as well as from communication between the physician and the nurses in front of the patient. These findings fully correlate with the findings in the research studies of Zhang et al. [56], Newell and Jordan [57], Nguyen et al. [58] and others. Ismail and Omar [59] dealt with the communication aspects between the patient and the healthcare professional in more depth, noting the importance of examining the suitability of the individual communication styles affecting patients’ satisfaction and their preference over other factors. According to the results of the study of these authors, the greatest influence on patient satisfaction lies in the communication style of the physician that emphasises decency, warmth and showing love and affection. The cultural aspects are also important because they open up another dimension of research. Bredart et al. [60] recommended the development of various communication strategies to increase patient satisfaction aimed at better communication between physician and patient, and the application of the various tools to obtain feedback to improve the communication forms. This fully corresponds to the results of the Lau [61] and Grayson-Sneed et al. [62] studies. The importance of researching communication forms is also confirmed by the study of Artati et al. [63], who also pointed out the different effects of verbal and nonverbal communication. It is relatively little studied in the patient satisfaction analysis. Effective physician–patient communication is an important clinical skill to build a physician–patient relationship. A good physician–patient relationship can increase work satisfaction and enhance patient self-confidence as well as a positive image of their health status that may affect the outcome of the disease. This is confirmed by the study by Naoum [64], who compared the benefits, the issues and the strategies for improving the physician–patient relationship. Wei et al. [65] also drew attention to the important interactions related to physician–patient communication and their benefits. According to the authors, physician–patient communication strongly interacts with patient satisfaction, and, thus, it also affects the patient’s risk perception. Hence, it supports the creation of greater trust between the patient and the physician, and this also affects the success of treatment. These consistent facts suggest that the physician–patient communication dimension has strong relations to several aspects related to the quality of healthcare and the patient’s health consequently. Numerous studies will be needed to improve the physician–patient communication processes, but as Boquiren et al. [66] mentioned, their successful implementation will require careful consideration of the objectives and the purpose of the measurement.

Our study found patient’s knowledge of the exchange of the work shifts of nurses in the department, the communication of the physician in front of the patient as if the patient was not present were not proven as the important indicators of patient satisfaction. It follows that the indicators directly related to its treatment are important for patients to a lesser extent with the processes for its provision. These findings fully correspond to the results of the research studies by Santuzzi et al. [17] and Bjertnaes et al. [28]. The authors emphasised the importance of the size of the research sample and the investigation of the reasons for patient dissatisfaction that are very important for managers and planners of the healthcare facilities. The work shifts, their occupancy, and the similar process activities are not directly linked to the perception of patient satisfaction nor associated with any expectations. The expectations are also recommended by the authors Bjertnaes et al. [28] and Marimon et al. [29], as they appear only very rarely in the evaluation systems. According to these authors, meeting expectations is the main linking mechanism between quality and patient satisfaction.

The results of the SEM analysis demonstrated that satisfaction with the quality of treatment provided (β = 0.538) and satisfaction with leaving the hospital (β = 0.547) were the factors that have a lower direct impact on the patient’s overall satisfaction with the healthcare provided. This is slightly contrary to many studies suggesting that the satisfaction of hospitalised patients is related to the quality of healthcare, diagnosis and treatment processes ([21,23,24,30,56]). This fact can also be explained by the results of the study by Farzianpour et al. [16], who considered patient satisfaction as a cognitive response influenced by various factors. They considered the determination of the factors influencing patient satisfaction as an indirect way to achieve real patient satisfaction. This author also emphasised the importance of understanding the expectations and the needs of patients, as did Bjertnaes et al. [28], Marimon et al. [29] and others. The evaluation of the quality of the provided treatment is also influenced by diagnoses and comorbidities that patients can manage differently. In this context, it is also important to examine the vulnerability of patients in relation to their comprehensive support needs. According to the research results, leaving the hospital was mostly associated with the standardised processes that patients did not perceive differently. These processes should also be connected to the connection of subsequent treatment in outpatient healthcare, in the other healthcare facilities depending on social status, age and diagnosis, respectively.

Written information for the patient about what he should do after their release from the hospital, the evaluation of the quality of the patient’s treatment in the hospital, together with informing the patient of the procedure in a case of a complaint about the quality of healthcare, were not significant determinants of patient satisfaction. Many research studies provide evidence of the importance of informing the patient not only in the dimension of assessing patient satisfaction but also to ensure the quality of the follow-up treatment in the outpatient sphere. This is also confirmed by the study of Yang et al. [67], in which the authors stated that complex medical patients experienced varying levels of concern and information needs after their release from the healthcare facility. The instructions for the patient on the next steps after their release from the healthcare facility should also motivate the patient to understand their role for a subsequent examination by a general practitioner. The other factors also played an important role in this respect, such as, for instance, availability of general practitioners in the region, clarity of instructions for release from the healthcare facility, geographical availability of healthcare (transport factor), social factors and so forth.

It will be necessary to activate the development and testing of the interventions to support post-hospitalisation healthcare that would address these issues. To do this, it is important to understand the patient’s state of health, understanding the role of general practitioners in the process of post-hospital and subsequent healthcare [67]. In the organisational context, medical staff play an important role in improving awareness of the processes [68]. Hellesø et al. [69] also emphasised the organisational context and its importance in the evaluation of the quality of information management in the healthcare facilities, but a level of OHL is also important. OHL aims to respond to patients’ health literacy needs by improving the health information and services provided and facilitating their understanding, access and application. Brega et al. [41] and Bremer et al. [42] called for insufficient research in this area, and they offered the appropriate criteria for monitoring and evaluating OHL.

Although our research confirmed that informing patients in writing about further treatment after release from the hospital as well as the procedures for a patient’s decision to complain about the quality of healthcare, were not the significant determinants of patient satisfaction, this does not mean that the healthcare facilities do not have to pay adequate attention to this process in the improvement in OHL. Patient satisfaction may change over time, and, therefore, it will be necessary to continuously monitor those aspects that have been identified as currently irrelevant to the evaluated patient satisfaction at a given time. The results in the satisfaction assessment may also vary depending on the type of hospital (public, private, specialised), as well as within the individual regions and the geographical distribution of the regions in the individual countries [7,43,70,71,72]. The information processes are influenced not only by the hospital’s information management but also by the information provider itself (physician, medical staff, nurses), the complexity of health information and whether the patient can understand it from different health and demographic aspects (age, education, diagnosis and so on) [73,74,75].

The least direct effect on patient satisfaction with inpatient care was confirmed in relation to hospital, department, room and board assessments (β = 0.233). The patients-responders evaluated the waiting time for room allocation from the moment the patient arrived at the hospital; the cleanliness of the hospital room and department, noise of medical staff at night. The patient satisfaction factor was not determined by choice of meal in the hospital or the night noise from other patients in the room. These findings do not correspond to the findings in the study by Kraska et al. [37], who examined the effect of hospital characteristics on patient satisfaction. All the analysed hospital variables had a significant effect on patient satisfaction in their study, but the differences in hospital satisfaction were found in the hospitals at the different locations. Here, too, the abovementioned geographical aspect and a barrier in comparing the results of patient satisfaction from a regional perspective were confirmed.

Warouw [76] recommend an examination of satisfaction with the hospital room in terms of patient satisfaction along with the quality of nursing service, which allows you to evaluate the other aspects, communicative or related to the treatment process. Persson et al. [77] had a different view on patient room evaluation in terms of patient satisfaction, and reported in their study that patients rate satisfaction with a hospital room based on how safe they feel in the hospital room and whether they can create a personal environment free of distractions. A private room also means a feeling of coziness and enhances treatment, but feelings of loneliness and isolation can also occur, which, in turn, can worsen the patient’s health. It is important to individually evaluate the patient’s preferences in relation to the diagnoses or treatment that they have to undergo in the hospital. This also draws attention to innovative patient accommodation planning that can enable the social benefits of dormitories to be maintained without compromising patient privacy and safety [78,79]. In addition to this parameter, patient design can also be affected by the design features of hospital rooms as well as the cross-cultural differences, as confirmed by the study by Devlin et al. [80].

These mentioned findings suggest that while primary patient satisfaction surveys can identify the preferential effects of factors in assessing satisfaction, there is a need to further investigate dissatisfied patients and the reasons for their dissatisfaction to identify the determinants that have the greatest impact on this level of dissatisfaction. As patient satisfaction is expressed by their cognitive response [16] influenced by many factors, it will still be problematic to standardise the indicators that would be most important and relevant in order to improve the hospital processes and to create an optimal comparative basis. A high level of individuality in approach to treatment, determined not only by the approach of physicians and healthcare professionals but also by the diagnoses requiring the various forms of diagnostic and treatment processes, will still define the boundaries of greater or lesser patient satisfaction and influence the overall outcome of their evaluation over time [16,17,19]. The socioeconomic and demographic characteristics of the patients themselves will also play an important role, as they will help create a comparison base for patients with domestic conditions and, thus, to set larger scales or smaller expectations of patients [18,20,22]. It follows that the issue of assessing patient satisfaction also has a strong psychological framework, and its knowledge allows managers and planners to actively develop appropriate strategies not only to improve patient satisfaction but also to monitor the effects of changes after taking appropriate measures.

The results of our research provide a valuable platform for policymaking, as well as for national and regional strategic health plan developers who need information on the quality of health care provided, the reasons for patient dissatisfaction and how to eliminate them. Knowledge of this information is also necessary for solving the issues related to the uneven demand for health care in the individual regions that may also result in a deterioration of health in the individual localities and so forth [36]. Many studies also point to caution in an interpretation of the findings from the comparisons of the different types of hospitals as an inconsistent comparative basis for patient satisfaction assessment may lead to the misleading illustrations of the differences in patient satisfaction [43]. This also draws attention to the role of social media in this field with hospitalisations and their importance not only for health care providers themselves but also for health policymakers, as confirmed by studies by Rai [34], Chakraborty and Church [30], Geletta [33], Alkazemi et al. [32] and others. Social networks can provide important information and, thus, complement research initiated by the research institutions or the health insurance companies, the health facilities and so on [35]. An important role in this direction is played by their periodicity so that it is also possible to measure and evaluate the effectiveness of the measures implemented by the hospital aimed at increasing the quality of healthcare and, thus, patient satisfaction over time [81]. In order for social media to be an active accepted part of systematic monitoring of patient satisfaction, it is necessary to carry out more research in this area and to evaluate the obstacles associated with this way of improving patient satisfaction—for instance, regularity and sufficiency of the number of patient reviews, willingness to communicate on social networks, the relevance of reviews and so forth. The increasing pressure on the efficiency of the healthcare systems in the individual countries will create additional opportunities to assess patient satisfaction, and their effective combination can significantly improve healthcare regardless of the geographical aspects as well as the aspects related to the type of the healthcare facility.

## 6. Conclusions

The patient satisfaction assessment has long been a proclaimed topic in both professional and research communities that supports the creation of a methodological platform and the initiation of new research aimed at revealing new determinants affecting the final assessment of patient satisfaction. The increasing pressures for higher efficiency of the healthcare facilities, as well as the sustainability of the healthcare systems, will also place higher demands on patient care and affect their loyalty to the healthcare facility. For this reason, various mechanisms are being created, and strategies for improving the healthcare quality for which a standardized platform and unification of the evaluated parameters cannot occur, but which set optimal parameters of healthcare quality, from which it is possible to set processes of its continuous improvement are being developed. In this process, the patient becomes an effective active indicator that can point out the deviations in the actual state from the desired or expected one. The issue of patient satisfaction is becoming increasingly multidisciplinary due to a deeper investigation into the causes of dissatisfaction and the search for ways to eliminate it. Satisfaction with the patient room can also have several dimensions, from technical to social, and all these points are aggregated in the final evaluation by the patient. For this reason, it is extremely important to continue to initiate new research with a multidisciplinary scope and to provide the health sector, the healthcare institutions, as well as the other actors in the healthcare system, with the most relevant information about the current state, its development and optimal solutions.

Our research was carried out with these intentions, the partial results of which are contained in this study. The aim of the study was to analyse and evaluate the determinants influencing the overall satisfaction of patients with inpatient healthcare in the conditions of the Czech Republic. The research sample consisted of 1425 patients with experience of inpatient care in the country. The results of the SEM model showed that selected factors, such as satisfaction with the hospital, department, room and board; satisfaction with medical staff; satisfaction with the quality of the treatment provided, and satisfaction with release from the hospital, have a positive effect on patient satisfaction with hospitalisation. Satisfaction with the medical staff was the most significant factor. On the other hand, satisfaction with the hospital, department, room and board had a significant effect, but with the least effect on satisfaction with hospitalisation.

The limitation of the research was the uneven distribution of the research sample within the individual regions of the Czech Republic, as it was not possible to influence the motivation of patients’ involvement in our research. Our research abstracted from the parameter of examining the equal satisfaction of patients in the regions within the Czech Republic, as the individual regions have different numbers of healthcare facilities with the different forms, sizes, specialisations, geographical locations within the cities and municipalities and so forth, that would have made the comparison of patient satisfaction between the regions of the Czech Republic irrelevant. The methodological aspects in the comparative analyses and the size of the research sample still remain a very important factor for the evaluation processes. The results of our study provide valuable information for policymakers as well as managers and planners in healthcare facilities. Actively and permanently improving patient satisfaction and the factors affecting it can reduce hospital operating costs, improve the patient experience and increase the efficiency of health resource allocation. Hospitals should strengthen the exchange of medical information between physicians and patients and improve the level of use of the health technologies in order to ensure better healthcare and a healthier population thus. The results of the patient satisfaction research defined areas for hospitals to improve the quality-of-service provision. However, it can take several years to achieve this improvement; not all hospitals structurally analyse changes in patient satisfaction over time. As a result, there is a lack of information from the patients’ point of view on the effectiveness of improvement programmes. This is also a challenge for follow-up research in this area.

The future research ambition of the authors is to compare patients’ attitudes according to demographic, social and economic characteristics. The authors expect further interesting findings. Furthermore, the comparison of the waves of Coronavirus in the 2019 (COVID-19) pandemic can also provide important results.

## Figures and Tables

**Table 1:** Validities and Reliabilities.

LVs	MVs	TV	FL	CA	CR	AVE
LV1: Satisfaction with hospital, department, room and board	MV_11	IV	0.727	0.847	0.775	0.535
	MV_13		0.697			
	MV_14		0.769			
LV2: Satisfaction with medical staff (nurses, physician’s expertise and other staff)	MV_21	IV	0.759	0.798	0.811	0.520
	MV_23		0.817			
	MV_24		0.629			
	MV_25		0.663			
LV3: Satisfaction with the quality of treatment provided	MV_31	IV	0.803	0.926	0.894	0.589
	MV_33		0.606			
	MV_34		0.836			
	MV_35		0.852			
	MV_36		0.831			
	MV_37		0.637			
LV4: Satisfaction with leaving the hospital	MV_41	IV	0807	0.847	0.734	0.581
	MV_42		0.714			
LV5: Satisfaction with inpatient care	MV_51	DV	0.908	0.902	0.919	0.792
	MV_52		0.922			
	MV_55		0.837			

Notes: LV—Latent variable; MV—Manifest variable; TV—Type of variable; IV—Independent variable; DP—Dependent variable; FL—Factor loading; CA—Cronbach alpha; CR—Composite Reliability; AVE—Average Variance Extracted. Source: own research

**Table 2:** Results of the suitability of the final SEM model.

The Goodness of Fit Measures	Absolute Fit Indices			Relative Fit Indices			Non-Centrality-Based Indices		
	CMIN/df	*p*-Value	GFI	NFI	IFI	TLI	CFI	RMSEA	RNI
Measurement Model	2.625	0.038	0.959	0.937	0.974	0.969	0.973	0.022	0.964
Structural Model	2.782	0.041	0.953	0.921	0.968	0.963	0.927	0.024	0.959
Criterion (Threshold values)	<5.0	<0.05	>0.95	>0.90	>0.95	>0.95	>0.95	<0.05	>0.95

Source: own research.

**Table 3:** Hypothesis evaluation.

H	Independent Variable (IV)	Regression Paths	β	SE	T	*p*-Value	Decision
H1	LV1: Satisfaction with hospital, department, room and board	LV1 † → LV5	0.233	0.039	6.689	0.000 *	Supported
H2	LV2: Satisfaction with medical staff (nurses, physician’s expertise and other staff)	LV2 † → LV5	0.597	0.041	16.974	0.000 *	Supported
H3	LV3: Satisfaction with the quality of treatment provided	LV3 † → LV5	0.538	0.025	20.363	0.000 *	Supported
H4	LV4: Satisfaction with leaving the hospital	LV4 † → LV5	0.547	0.043	11.721	0.000 *	Supported

Note: H—Hypotheses; β—Standardised Regression weights; † = Predictor; SE—Standard error; T—Student test criterion; * *p* < 0.05. Source: own research.

## Data Availability

The data presented in this study are available on request from the corresponding author. The data are not publicly available due to [Data can be provided only in the Czech language at the request of the corresponding author.].

## References

[1] Jennings B.M., Heiner S.L., Loan L.A., Hemman E.A., Swanson K.M. (2005). What Really Matters to Healthcare Consumers. JONA J. Nurs. Adm..

[2] Verulava T., Sibashvili N. (2015). Accessibility to psychiatric services in georgia. Afr. J. Psychiatry.

[3] Verulava T., Jincharadze N., Jorbenadze R. (2017). Role of primary health care in re-hospitalization of patients with heart failure. Georgian Med. News.

[4] Gavurova B., Rigelsky M., Ivankova V. (2020). Perceived health status and economic growth in terms of gender-oriented inequalities in the OECD countries. Econ. Sociol..

[5] Ucieklak-Jeż P., Bem A. (2020). Does “Rural” Always Mean the Same? Macrosocial Determinants of Rural Populations’ Health in Poland. Int. J. Environ. Res. Public Health.

[6] Bem A., Siedlecki R., Prędkiewicz P., Gazzola P., Ryszawska B., Ucieklak-Jeż P. (2019). Hospitals’ Financial Health in Rural and Urban Areas in Poland: Does It Ensure Sustainability?. Sustainability.

[7] Preędkiewicz P., Bem A., Ucieklak-Jezż P., Siedlecki R., Jajuga K., Locarek Junge H., Orlowski L.T., Staehr K. (2019). Public or Private? Which Source of Financing Helps to Achieve Higher Health System Efficiency?. Contemporary Trends and Challenges in Finance.

[8] Lieskovska V., Megyesiova S., Petrovcikova K. Marketing as a tool of health care support. Proceedings of the 12th International Scientific Conference on Reproduction of Human Capital—Mutual Links and Connections (RELIK).

[9] Megyesiova S., Lieskovska V. (2018). Analysis of the Sustainable Development Indicators in the OECD Countries. Sustainability.

[10] Megyesiova S., Lieskovska V. (2019). Premature Mortality for Chronic Diseases in the EU Member States. Int. J. Environ. Res. Public Health.

[11] Gavurova B., Kovac V., Khouri S. (2020). Purpose of patient satisfaction for efficient management of healthcare provision. Pol. J. Manag. Stud..

[12] Riklikiene O., Didenko O., Ciutiene R., Daunoriene A., Ciarniene R. (2020). Balancing nurses’ workload: A case study with nurse anaesthetists and intensive care nurses. Econ. Sociol..

[13] Jakubowska A., Bilan S., Werbiński J. (2021). Chronic diseases and labour resources: “Old and new” European Union member states. J. Int. Stud..

[14] Sopko J., Kočišová K. (2019). Key Indicators and Determinants in the Context of the Financial Aspects of Health Systems in Selected Countries. Adiktologie.

[15] Briestenský R., Ključnikov A. (2019). Identification of the Key Factors for Successful Hospital Management in Slovakia. Adiktologie.

[16] Farzianpour F., Byravan R., Amirian S. (2015). Evaluation of Patient Satisfaction and Factors Affecting It: A Review of the Literature. Health.

[17] Santuzzi N.R., Brodnik M.S., Rinehart-Thompson L., Klatt M. (2009). Patient Satisfaction: How Do Qualita-tive Com-ments Relate to Quantitative Scores on a Satisfaction Survey?. Qual. Manag. Health Care.

[18] Yin T., Yin D.-L., Xiao F., Xin Q.-Q., Li R.-L., Zheng X.-G., Yang H.-M., Wang L.-H., Ding X.-Y., Chen B.-W. (2019). Socioeconomic status moderates the association between patient satisfaction with community health service and self-management behaviors in patients with type 2 diabetes. Medicine.

[19] Hu L., Ding H., Liu S., Wang Z., Hu G., Liu Y. (2019). Influence of patient and hospital characteristics on inpatient satisfaction in China's tertiary hospitals: A cross-sectional study. Health Expect..

[20] Chumbler N.R., Otani K., Desai S.P., Herrmann P.A., Kurz R.S. (2016). Hospitalized Older Adults’ Patient Satisfaction: Inpatient care experiences. SAGE Open.

[21] Carr A.B., Nicolau D.P. (2017). The challenges of patient satisfaction: Influencing factors and the patient—Provider relationship in the United States. Expert Rev. Anti-Infect. Ther..

[22] Hamka S.W. (2018). Effect of Service Quality and Customer Satisfaction Patients in General Hospitals of Makassar City Region. J. Phy..

[23] Mitropoulos P., Vasileiou K., Mitropoulos I. (2018). Understanding quality and satisfaction in public hospital services: A nationwide inpatient survey in Greece. J. Retail. Consum. Serv..

[24] Eisfeld H., Bauer F., Dubois C., Schmidt T., Kastrati K., Hochhaus A., Hübner J. (2019). Importance of and Satisfaction with Information about Their Disease in Cancer Patients. J. Cancer Educ..

[25] Gonzalez-Zacarias A.A., Mavarez-Martinez A., Arias-Morales C.E., Stoicea N., Rogers B. (2016). Impact of Demographic, Socioeconomic, and Psychological Factors on Glycemic Self-Management in Adults with Type 2 Diabetes Mellitus. Front. Public Health.

[26] Su R., Cai L., Cui W., He J., You D., Golden A. (2016). Multilevel Analysis of Socioeconomic Determinants on Diabetes Prevalence, Awareness, Treatment and Self-Management in Ethnic Minorities of Yunnan Province, China. Int. J. Environ. Res. Public Health.

[27] Tates K., Antheunis M.L., Kanters S., Nieboer T.E., Gerritse M.B. (2017). The Effect of Screen-to-Screen Versus Face-to-Face Consultation on Doctor-Patient Communication: An Experimental Study with Simulated Patients. J. Med Internet Res..

[28] Bjertnaes O.A., Sjetne I.S., Iversen H.H. (2011). Overall patient satisfaction with hospitals: Effects of patient-reported experiences and fulfilment of expectations. BMJ Qual. Saf..

[29] Marimon F., Gil-Doménech D., Bastida R. (2017). Fulfilment of expectations mediating quality and satisfaction: The case of hospital service. Total. Qual. Manag. Bus. Excel..

[30] Chakraborty S., Church E.M. (2021). Patient hospital experience and satisfaction on social media. Int. J. Qual. Serv. Sci..

[31] Chakraborty S., Church E.M. (2020). Social media hospital ratings and HCAHPS survey scores. J. Health Organ. Manag..

[32] Alkazemi M.F., Bayramzadeh S., Alkhubaizi N.B., Alayoub A. (2019). The physical environment and patient satisfaction ratings on social media: An exploratory study. Facilities.

[33] Geletta S. (2018). Measuring patient satisfaction with medical services using social media generated data. Int. J. Health Care Qual. Assur..

[34] Rai T.S. (2019). Social media use and mental health. Science.

[35] Rastegar-Mojarad M., Ye Z., Wall D., Murali N., Lin S. (2015). Collecting and Analyzing Patient Experiences of Health Care from Social Media. JMIR Res. Protoc..

[36] Liu M., Hu L., Guo R., Wang H., Cao M., Chen X., Liu Y. (2021). The Influence of Patient and Hospital Characteristics on Inpatient Satisfaction at Beijing District-Level Hospitals. Patient Prefer. Adherence.

[37] Kraska R.A., Weigand M., Geraedts M. (2016). Associations between hospital characteristics and patient satisfaction in Germany. Health Expect..

[38] Mann R.K., Siddiqui Z., Kurbanova N., Qayyum R. (2015). Effect of HCAHPS reporting on patient satisfaction with physician communication. J. Hosp. Med..

[39] Davidson K., Shaffer J.A., Ye S., Falzon L., Emeruwa I.O., Sundquist K., Inneh I.A., Mascitelli S.L., Manzano W.M., Vawdrey D.K. (2016). Interventions to improve hospital patient satisfaction with healthcare providers and systems: A systematic review. BMJ Qual. Saf..

[40] Weidmer B.A., Brach C., Slaughter M.E., Hays R.D. (2012). Development of Items to Assess Patients’ Health Literacy Experiences at Hospitals for the Consumer Assessment of Healthcare Providers and Systems (CAHPS) Hospital Survey. Med. Care.

[41] Brega A.G., Hamer M.K., Albright K., Brach C., Saliba D., Abbey D., Gritz R.M. (2019). Organizational Health Literacy: Quality Improvement Measures with Expert Consensus. Health Lit. Res. Pract..

[42] Bremer D., Klockmann I., Jaß L., Härter M., von dem Knesebeck O., Lüdecke D. (2021). Which criteria characterize a health literate health care organization?—A scoping review on organizational health literacy. BMC Health Serv. Res..

[43] Siddiqui Z.K., Wu A.W., Kurbanova N., Qayyum R. (2014). Comparison of Hospital Consumer Assessment of Healthcare Providers and Systems patient satisfaction scores for specialty hospitals and general medical hospitals: Confounding effect of survey response rate. J. Hosp. Med..

[44] Jöreskog K.G. (1970). A general method for estimating a linear structural equation system. ETS Res. Bull. Ser..

[45] Fan X., Thompson B., Wang L. (1999). Effects of sample size, estimation methods, and model specification on structural equation modeling fit indexes. Struct. Equ. Model. A Multidiscip. J..

[46] Hooper D., Coughlan J., Mullen M.R. (2008). Structural equation modelling: Guidelines for determining model fit. Electronic J. Bus. Res. Methods.

[47] Kaiser H.F. (1974). An index of factorial simplicity. Psychometrika.

[48] Byrne B.M. (2001). Structural Equation Modeling With AMOS, EQS, and LISREL: Comparative Approaches to Testing for the Factorial Validity of a Measuring Instrument. Int. J. Test..

[49] Hair J.F., Hauff S., Hult G.T.M., Richter N.F., Ringle C.M., Sarstedt M. A Primer on Partial Least Squares Structural Equation Modeling.

[50] Lu J., Ren L., Zhang C., Wang C., Ahmed R.R., Streimikis J. (2020). Corporate social responsibility and employee behavior: Evidence from mediation and moderation analysis. Corp. Soc. Responsib. Environ. Manag..

[51] Martínez-López F.J., Gázquez-Abad J.C., Sousa C.M.P. (2013). Structural equation modelling in marketing and business research: Critical issues and practical recommendations. Eur. J. Mark..

[52] Shah R., Goldstein S.M. (2005). Use of structural equation modeling in operations management research: Looking back and forward. J. Oper. Manag..

[53] Olsson U.H., Foss T., Troye S.V., Howell R.D. (2000). The Performance of ML, GLS, and WLS Estimation in Structural Equation Modeling Under Conditions of Misspecification and Nonnormality. Struct. Equ. Model. A Multidiscip. J..

[54] Bentler P.M. (1990). Comparative fit indexes in structural models. Psychol. Bull..

[55] Ahmed R.R., Hussain S., Pahi M.H., Usas A., Jasinskas E. (2019). Social Media Handling and Extended Technology Acceptance Model (ETAM): Evidence from SEM-based Multivariate Approach. Transform. Bus. Econ..

[56] Zhang L., Chen H., Li M., Wang J., Xue C., Ding T., Liu Y., Nong X. (2016). Factors influencing inpatients’ satisfaction with hospitalization service in public hospitals in Shanghai, People’s Republic of China. Patient Prefer. Adherence.

[57] Newell S., Jordan Z. (2015). The patient experience of patient-centered communication with nurses in the hospital setting: A qualitative systematic review protocol. JBI Database Syst. Rev. Implement. Rep..

[58] Nguyen J., Hunter J., Smith L., Harnett J.E. (2021). Can We All Speak the Same ‘Language’ for Our Patients’ Sake? Feedback on Interprofessional Communication and Related Resources. Glob. Adv. Health Med..

[59] Ismail F., Perlis U.M., Omar B., Malaysia U.S. (2018). Kesan Gaya Komunikasi Doktor Perubatan Terhadap Kepuasan Pesakit (The Effects of Physician Communication Style on Patient Satisfaction). J. Komunikasi Malays. J. Commun..

[60] Brédart A., Bouleuc C., Dolbeault S. (2005). Doctor-patient communication and satisfaction with care in oncology. Curr. Opin. Oncol..

[61] Lau F.L. (2000). Can communication skills workshops for emergency department doctors improve patient satisfaction?. J. Accid. Emerg. Med..

[62] Grayson-Sneed K.A., Dwamena F.C., Smith S., Laird-Fick H.S., Freilich L., Smith R.C. (2016). A questionnaire identifying four key components of patient satisfaction with physician communication. Patient Educ. Couns..

[63] Artati R.D., Pasinringi S.A., Jafar N. The Effect of Doctors-Patients' Verbal and Non verbal Communication on Patients' Satisfaction in Outpatient Installation of Mother and Child Centre RS Dr. Wahidin Sudirohusodo Makassar. Proceedings of the International Conference on Healthcare Service Management (ICHSM).

[64] Naoum S. (2018). Doctor-Patient Communication: Benefits, problems and strategies for improvement. Sci. Chron..

[65] Wei D., Xu A., Wu X. (2019). The mediating effect of trust on the relationship between doctor–patient communication and patients’ risk perception during treatment. PsyCh J..

[66] Boquiren V.M., Hack T.F., Beaver K., Williamson S. (2015). What do measures of patient satisfaction with the doctor tell us?. Patient Educ. Couns..

[67] Yang S.C., Zwar N., Vagholkar S., Dennis S., Redmond H. (2009). Factors influencing general practice follow-up attendances of patients with complex medical problems after hospitalization. Fam. Pract..

[68] Willems J., Ingerfurth S. (2018). The quality perception gap between employees and patients in hospitals. Health Care Manag. Rev..

[69] Hellesø R., Lorensen M., Sorensen L., Norman L., Bang K. (2006). Management of information between two nursing contexts. Stud. Health Technol. Inform..

[70] Semyonov-Tal K. (2021). Complaints and Satisfaction of Patients in Psychiatric Hospitals: The Case of Israel. J. Patient Exp..

[71] Mainous A.G., Diaz V.A., Everett C.J., Knoll M.E. (2011). Impact of Insurance and Hospital Ownership on Hospital Length of Stay Among Patients With Ambulatory Care-Sensitive Conditions. Ann. Fam. Med..

[72] Andemeskel Y.M., Elsholz T., Gebreyohannes G., Tesfamariam E.H. (2019). Patient satisfaction with peri-operative anesthesia care and associated factors at two National Referral Hospitals: A cross sectional study in Eritrea. BMC Health Serv. Res..

[73] Showers N., Simon E.P., Blumenfeld S., Holden G. (1995). Predictors of Patient and Proxy Satisfaction with Discharge Plans. Soc. Work. Health Care.

[74] Perry L., Middleton S. (2011). An investigation of family carers' needs following stroke survivors' discharge from acute hospital care in Australia. Disabil. Rehabil..

[75] Brühwiler L.D., Beeler P.E., Böni F., Giger R., Wiedemeier P.G., Hersberger K.E., Lutters M. (2019). A RCT evaluating a pragmatic in-hospital service to increase the quality of discharge prescriptions. Int. J. Qual. Health Care.

[76] Warouw H.J. (2017). The Quality of Nursing Service and Patients’ Satisfaction In The Inpatient Room of Pancaran Kasih Gmim Hospital Manado. Int. J. Health Med. Curr. Res..

[77] Persson E., Anderberg P., Ekwall A.K. (2014). A room of one′s own—Being cared for in a hospital with a single-bed room design. Scand. J. Caring Sci..

[78] Tzeng H.-M., Yin C.-Y. (2008). The Extrinsic Risk Factors for Inpatient Falls in Hospital Patient Rooms. J. Nurs. Care Qual..

[79] Kleefstra S.M., Zandbelt L.C., De Haes H.J.C.J.M., Kool R.B. (2015). Trends in patient satisfaction in Dutch university medical centers: Room for improvement for all. BMC Health Serv. Res..

[80] Devlin A.S., Andrade C.C., Carvalho D. (2015). Qualities of Inpatient Hospital Rooms: Patients' Perspectives. HERD Health Environ. Res. Des. J..

[81] Raven M.C., Gillespie C.C., Dibennardo R., Van Busum K., Elbel B. (2011). Vulnerable Patients' Perceptions of Health Care Quality and Quality Data. Med. Decis. Mak..

